# Insecurities of Women Regarding Breast Cancer Research: A Qualitative Study

**DOI:** 10.1371/journal.pone.0081770

**Published:** 2013-12-02

**Authors:** Marion Habersack, Gero Luschin

**Affiliations:** 1 Office of the Vice Rector for Teaching and Studies, Medical University Graz, Graz, Austria; 2 Womens Health Association Graz, Graz, Austria; National Taiwan University, College of Medicine, Taiwan

## Abstract

**Objectives:**

Only 1.2%–11% of all potential study participants participate in cancer studies. Low participation rates can result in bias or in a failure to obtain data saturation. Subject-scientific psychology assumes that reasons for acting are based on individual premises. The objective of this study was to render reproducible individual reasons of female breast cancer patients to participate or not participate in breast cancer studies using a qualitative approach.

**Methods:**

Problem-based interviews were conducted with female breast cancer patients. The selection of interview partners continued until theoretical data saturation was achieved.

**Results:**

As main arguments against participation emotional overload and too many medication side-effects were stated. Improvement of health-related values, long-term protection and comprehensive follow-up exams were stated as arguments for participation. Trust in the attending physician was mentioned as influencing both participation and non-participation.

**Conclusions:**

A significant influential factor determining willingness to participate in studies was one's contentment with patient-physician communication. In order to guarantee an adequate patient decision-making process, keeping existing standards for patient briefings is absolutely mandatory.

## Introduction

It is known that only 1.2%–11% of all potential study participants actually participate in cancer studies [1]–[4]. A low participation rate can lead e.g. to bias and can result in a clinical effectiveness being shown as not significant [5]. As outlined in earlier work, only few studies about the reasons for and against participation in medicinal breast cancer research have been published [1]. As possible reasons against participation the literature mentions, among other things, the fear of side effects, skepticism towards clinical studies, the feeling of becoming an experiment by participating or the desire for other treatment [2], [4], [6], [7]. As reasons for participation the literature mentions e.g. the feeling of not being able to reject the suggestions of physicians, satisfaction with the received information and/or knowledge and qualification of physicians, no placebo and acceptance of randomization [8]–[11]. However, subject-scientific psychology assumes that reasons for acting are based on individual premises. Therefore, research regarding behaviors is not limited to cause and effect principles, but is expanded to reasoning from a theoretical view [12]. These reasons or assumptions can be reproduced without outside attributions through mutual communication [13]. The aim of the present investigation was to collect individual reasons of female breast cancer patients for participation or non-participation in cancer studies using a qualitative approach.

## Methods

This qualitative research was conducted between May and December 2011. Women with early breast cancer who either participate or do not participate in a placebo-controlled breast cancer study were selected as interview partners. The breast cancer study is a comparative investigation of an osteoporosis medication (Denosumab) with a placebo during aromatase-inhibitor therapy (EudraCT number 2005-005275-15). This drug is used for treatment and prevention of osteoporotic or metastatically conditioned bone fractures in breast cancer patients.

According to the recommendations for qualitative social research, an open approach was chosen for the determination of individual reasons. Specifically, we used problem-focused structured interviews [14]–[16]. This method allowed enough freedom to follow-up and expands on reasons for addressed items. In this manner it is possible to collect articulated reasoning with a higher degree of detail [17].

### Ethics Statement

Approval by the Ethics Commission was obtained (Ethikkommission des Landes Steiermark, Amt der Steiermärkischen Landesregierung). The individual persons in this manuscript have given written informed consent.

### Development of categories and leading question

Based on the literature search, three categories were developed (Table 1):

**Table 1:** Category Development.

Category	Description	Standard Examples and References
Person-related Reasons	This category comprises a broad spectrum of reasons that relate back to the person. These include health, psycho-social and socio-demographic reasons.	Health condition [8], [11], [22]–[24], [29], altruism [11], [24], [30], frequent mental examination of the topic of breast cancer [11], [23], feeling of becoming an experiment oneself [30], age [11], [23]
Study-related Reasons	This category comprises reasons that relate to the study protocol, as well as reasons that directly relate to the medication treatment.	Randomization [24], [30], placebo [8], time spent [11], [22], [24], [29], [30], interval between surgery and recruiting [8], willingness to be treated with the study medication [11], [24], [30], side effects [11], [22]–[24], [30]
Physician-related Reasons	This category contains reasons that relate to the passed on information in the context of the briefing on the study by the physician and the decision to participate.	Extent of information during physician consultations [9], [11], conviction that physicians have to take over the decision making [9], [30], fear of losing their own decision making [9], [30]

- Person-related reasons (health, psycho-social and socio-demographic)
- Study-related reasons (study protocol and treatment-related)
- Physician-related reasons (information and decision-related)

Leading interview questions were developed based on the three categories. After performing a pilot interview, one question (regarding the physician's role in patient briefing) was worded in more general terms in order to lower the inhibition threshold when answering. The questions served as conversation support. In this sense, the sequence of the questions did not have to be followed consistently, but could be adjusted to the respective interview situation [18]. (See Text S1)

### Data Collection

For inclusion in our study, women must meet the following criteria: a) have a non-metastasizing breast cancer treated with aromatase-inhibitor therapy, and b) had been offered participation in a study comparing an osteoporosis medication (i.e. the breast cancer medication study) with a placebo. Women were recruited until theoretical data saturation was obtained. Theoretical data saturation means, to have good reason to act on the assumption that one has captured everything important. In relation to the examined phenomenon (in the concrete study along the deductively established categories), “saturation” of the analysis means that additional individual analyses would not result in new phenomenon structures/interpretive models/reaction typologies. We reached this saturation by collecting data until no new information was achieved and data began to replicate [19]. After each interview we compared the concepts. In this way data collection was conducted like an iterative process for each interview.

A balanced proportion was sought between interview partners who had participated in the breast cancer medication study and those who had not. Potential interview partners were identified through a cancer registry and were contacted by telephone. During this first contact, the use and purpose of the planned interview were explained and anonymity of all data was assured. With the consent of the interview partners, the conversations were tape-recorded. Additionally, post-scripts were recorded after each interview, in which impressions and distinctive features were noted [18]. In one of the interviews, no consent for tape-recording was given. In this instance the conversation was recorded in keywords.

### Data Processing and Evaluation

The tape recordings were transcribed with the aid of the f4-media 1.0 program (Dresing & Pehl, Marburg, Germany) and following the transcription rules of Mayring [20]. The non-taped interview was summarized in a memory protocol. The coding organization was performed on a data-processing base with the MAXQDA 10 program (VERBI, Berlin, Germany). The interview evaluations were performed following structured content analysis rules, according to Mayring [21]. Additionally, the study participants group and the non-participants group were subjected to a thematically comparative analysis.

## Results

In total, 22 women with an age between 50 and 79 years were interviewed, 10 of them were breast cancer medication study participants and 12 were non-participants. On average, the interviews took 22 minutes to complete. There were no substantial differences between participants and non-participants with regards to age, anti-hormone medication, or family history of cancer. Fourteen women were briefed about the opportunity to participate in the study during 2008–2009, and eight during 2010–2011. For 12 women, there were less than three months between breast surgery and the briefing regarding the opportunity to participate in the study (see Table 2).

**Table 2:** Overview of the 22 Interview Partners.

		P	NP
		(n = 10)	(n = 12)
Age	50–64 years	6	6
	65–79 years	4	6
Medication	Anastrozol (Arimidex)	5	7
	Letrozol (Femara)	5	4
	Tamoxifen	0	1
Family History of Cancer	Yes	8	9
	No	1	0
	No Answer	1	3
Time of Briefing about the Study	2008–2009	8	6
	2010–2011	2	6
Time between Surgery and Briefing about the Study	≤3 Months	6	6
	3.1–6 Months	3	3
	>6.1 Months	1	3

P  =  Participants, NP  =  Non-participants

It was possible to keep the generated category system during the examination of the interview material. Inductively, no further categories resulted.

The following reaction typologies were established:

In the category “person-related reasons”:

- Personal benefit (by participating in a study, the interview partners expect, among other things, better follow-up care, more detailed and exact consultation, closer contact with medical personnel)
- Altruism (as arguments for participating in a study, the benefit for research and future patients are referred to)
- Personal burden (as decisive for not participating in a study, the interview partners mention emotional stress, side effects, the categorization as test subject)

In the category “study-related reasons”:

- Attitudes regarding medication (the fear of side-effects, skepticism with regards to medication and unnecessary stress are listed as reasons for not participating in studies)
- Study protocol (placebo as burden and important reason for not participating)

In the category “physician-related reasons”:

- Trust in the physician (the interviewed women define trust in the physicians attending them/ informing them about the study as decisive for their decision to participate/not participate in a study)
- Collecting other opinions (the opinion of persons who are not involved in the study and who are trusted is mentioned as decision help for/against participating
- Information during the briefing (extensive, comprehensive and comprehensible briefing und trust in the physician are emphasized as important elements in the decision process)

### Person-related reasons

Regarding the question of personal reasons for participating or not participating in the study, personal benefit in the sense of health improvement or long-term protection, altruistic intentions, and personal burdens through study participation were mentioned.

#### Personal benefit: Long-term protection

Six of 10 participants indicated that an additional or primary motivation for their participation in the study was better follow-up care. They specifically mentioned that they wanted to be able to enjoy other current and more detailed medical examinations as well as extensive briefings.

*I usually have a rather extensive conversation with the physician and I just feel very well taken care of. And I believe, if I weren't participating in the study, I don't know if it would be as precise and personal with the follow-up care. (…), (…) it just simply really is a reassurance. Yes; and a good reassurance for me that it is right. Of course I am examined regarding my spine every six months; so that is automatically better. Otherwise I would not receive that because they are not authorized by the health insurance, except if they are absolutely necessary.*

One interview partner emphasized contact with the medical staff, including after the study was completed as additional protection.

*And what one may add to this—what I seem to read from it or the information that I have received is that even after the therapy is finished or the study is finished, up to five years some kind of follow-up care is carried out or there is also simply contact with the conducting medical person who then really asks and follows up annually how I am doing and what is going on now. And that is nice, too, that you don't just get the feeling, it's done now and now you fall into a void. Because it is not like you would say it is a topic that is finished then, but this will always stay with me.*

#### Third-party benefit/altruism

Altruistic intentions were mentioned as further motivations for participating in the study, that is, one's participation was to benefit others. Seldom was the well-being of other patients mentioned in this context, but research in general was considered a motivation to participate by half of the participants.

*…because it doesn't hurt; and if it is for research, I agree.*

Half of the non-participants expressed a troubled conscience with regard to future patients. Twice, mention was made regarding a troubled conscience regarding the attending physician.

*And I said, no, I really don't want to (softly) (,) and somehow it's something; for I have a bad conscience regarding the other women, because I think to myself, it actually benefits more the women after me. But then … you have a certain amount of selfishness and say no.*

#### No personal benefit: Personal burden

Ten of twelve women perceived participating in the study as a personal burden at the time when the study was explained to them. Two aspects were defined as straining: a) personal burden in the sense of emotional stress and b) stress in the sense of too many side effects. (The latter is explained in more detail in the study-related reasons category.)

*And I said that is just too much for me at the moment. I can't cope anymore. That was actually the real reason why I said no at the time.*

Several women referred to their diagnosis of cancer, which was a shock to them that they still had to work through. Others also discussed the physical side effects of the surgery (like scars) and of chemo- and radiation therapies (like hair loss), which were interpreted as a great psychological stressors. The interview partners agreed that during the follow-up program, time and rest were necessary and participation in the study would have meant an additional burden.

*One has passed this surgery and then has … (sighing) the head full with so many things. And then I would really like to be left alone. I don't want anything else to do with it anymore. And I believe, many think this way, because I am in contact with many of them and they say basically the same thing: I want to be left alone now, I have to somehow find my calm…*

Half of the participants mentioned that at the time when the study was explained to them, the shock regarding the cancer diagnosis had already been overcome, and that they were therefore able to once again look positively towards the future. They saw this as a further reason for study participation. The presence of this positive attitude was viewed as prerequisite for participation in such a medicinal study.

*I also think the time factor is important—so, right then, when the doctor addresses it, that I myself am in a good condition, that I am already doing well psychologically, that I have a positive attitude there and that my general physical condition is good too—because I think that, certainly, it simply is a prerequisite to be open to such studies.*

Six interview partners expressed their uneasiness about seeing themselves as part of a test series, an experiment. Three of these six persons were non-participants, who at the time of the briefing about the study were not yet emotionally ready to deal with something new, and uttered the desire for a respite.

*…then there was this; that I had radiation since February and then I just wanted to leave that behind me. And simple for me (,) I just wasn't in the composure, that I would have dealt with the whole thing anyways (!) and for me it was then simply (to be understood) as guinea pig.*

### Study-related reasons

The attitudes towards medications in general and to the study medication in particular differed considerably between participants and non-participants and substantially influenced their decisions to participate or not participate in the study. Though study protocol-specific reasons (like randomization, placebo, etc.) were mentioned, in most cases they were not decisive for participation.

#### Attitudes regarding medication

Half of the non-participants said they did not like to take medications, regardless of whether they were pills or injections. They indicated that they only took medications when it was really necessary because of sickness. The same women also voiced concerns with the study medication regarding certain side effects, especially when potential side effects already existed as symptoms of their own medical history.

*I am already almost blind as it is, I think. I already need reading glasses, 3.5 or three diopters. So, if that still gets worse or what …*

The conviction that each medication, and consequently also the study medication, can have side effects was prevalent in half of the interview partners. The non-participants considered this an unnecessary stress for their body and listed this stress as a primary reason for not participating in the study. In contrast, the participants did not even want to think of potential side effects. They were of the opinion that if the study medication was not going to do them any good they would sense it, and would be able to drop out of participating in the study.

*When it says there, there can be this or that in there, I mean, when I take my medications, I have the same problem. These ones aren't good for the stomach; the others aren't good for that. Then you wouldn't be allowed to do anything. So, I don't worry about it.*

#### Study protocol

The course and duration of the study, as well as randomization and placebo, were important items for the interview partners, but in most cases not crucial in their decision to participate or not participate in the study. Four non-participants as well as four participants viewed the placebo as a burden. For them it would be better to conduct medication studies without such a placebo. In spite of this skepticism towards the placebo, there were women who showed understanding of the use of the placebo. Some of the participants mentioned the coordination of the study appointments with the appointments of their follow-up program. This was not described as another reason for participation, but as positive “side effect” of the study.

### Physician-related reasons

#### Trust in the physician

For 10 women, trust in the briefing physicians was essential for participation in the study. The interview partners who had placed great trust in the attending physicians rarely voiced concerns regarding possible negative consequences of the study medication.

*No, I have to say, I only read that afterwards (handout on the study in question). I mean, I trusted the physician completely in this.*

They had trust in the physician and, thus, also in the further care. They partially viewed the study as a personal recommendation that could not hurt their health.

*A deciding factor for me, for the study, certainly was mainly the great trust in my physician. In my physician, in the treatment variation that he has suggested to me And that was certainly worth consideration, that I thought he certainly would not impose something on me that is negative for me and I was simply sure that it is a good thing.*

#### Collecting other opinions

One-fourth of the interviewed women indicated that they had asked physicians not involved in the study for their input. They listed great trust in these persons as the reason for doing so. In one case, a hospital nurse was shown this trust.

*Then I went to my gynecologist anyway and asked him once again what he thought of it. And then I also asked a doctor I didn't know, and they rather encouraged me then discouraged me. (That's why I went to two other doctors), because I think it's always better, two … (smiling) (,) well, yes, with several opinions you can still be unsure. But I rather ask for more opinions, but from those who I know and who I trust.*

Information on the internet was used by only one interview partner as a decision base for her participation in the study. One could formulate the hypothesis that a higher age could be an essential factor for not utilizing the internet. In contrast, the interview memos indicate that almost half of the interview partners make use of this new medium. The internet is mainly used for the communication via email and less for the gathering of information. Some women feared to find “bad news” searching the web about breast cancer.

*…also can research a little, perhaps also on the internet, though I don't like to do that so much. Of course I did it before, but. … I talked with my doctor today anyhow. He also says that, most of the time, those who write are those who have had negative experiences; where the surgery failed. That went wrong or this or that. With the others it is not like they sit down and just write something. That's how it is. And that's why I stopped that really fast again in order to investigate how the people fared or how the people are doing. That makes for a heavy mind. I don't need that.*

#### Information during the briefing

When responding to the question related to how they remember their physician's briefing regarding the study, more than half of the interview partners referred to the extent and the manner of sharing information. The participants described the briefing predominantly with the words “extensive”, “comprehensive” and “comprehensible”. In contrast, the non-participants indicated more short briefings.

*…he (the physician) said, yes, that, that, that, that we have done now and we take the Arimidex now … for five years and then he gave me the thing (the breast cancer medication study. He said, do you have the material already? I said, no, I have never seen this before. He put it in my hand. Read through this, he said, and then I should sign it and bring it. And … then I read through that at home and, and … I just wasn't ready. And perhaps also that it was simply put in my hand and they said I should read it. I just mean …, insofar as that there really wasn't any conversation.*

Here again, the interview partners articulated the importance of trust in the physicians. The extent and the accuracy of the information shared during the briefing were indispensable for the further trusting relationship between patient and physician.

*But I don't know when he tells me afterwards there also was an alternative, whether I could once again place my trust in the doctor. So, I believe it is really important when he tells me everything.*

## Discussions

The present qualitative investigation shows a broad spectrum of reasons why women participate or reject participation in medication studies. As a main argument against participating, the interview partners mentioned diverse stresses. Stresses in the sense of emotional stress at the time of briefing, or stresses in the sense of too many side effects of the study medication. Beyond that, the majority of non-participants generally viewed the taking of medications with great skepticism: medications were only to be taken when clear indications for them were present, meaning certain health reasons. Accordingly the probability of being willing to participate in the study was associated with worse clinical values. Loehberg et al. obtained similar findings in 2010, where for 42% of 172 women with breast cancer, an inconspicuous result in their breast examination was the reason for an unwillingness to participate in a medicinal study [22].

The effect of age on study participation has been discussed in the literature. For Mandelblatt et al. (2005) and Rondanina et al. (2008) the willingness to participate in a breast cancer study was significantly lower among older women [11], [23]. This was mostly ascribed to the distance between the participants' residence and the place where the study was conducted. Three of our interview partners (65–79 years old) listed their age as a decisive reason for not participating. They thought themselves too old, and justified their non-participation with the lack of personal benefit.

Conversely, some interview partners listed personal benefit and long-term protection as arguments for participating in the study. Personal benefit was in the form of improvement or at least maintenance of health and illness-related values, and long-term protection was in the sense of continued and comprehensive follow-up examinations. Furthermore, participation was justified by several interview partners with the altruistic argument that they were doing something good for research. It is worth mentioning that in the case of non-participation, a troubled conscience regarding other patients was expressed. Altschuler & Somkin (2005) arrived at partially similar findings; in their study, almost half of the 23 non-participating women mentioned their troubled conscience, lack of altruism and lack of contribution to the research effort during their interview [24].

An additional factor for participating in the study was trust in the attending physicians. This trust is to be taken as a possible indication for whether, how, where, and to what extent the interview partners gathered information regarding the study. The extent and accuracy of the information shared during the briefing were listed as reasons for participation in a broader sense. If the extent of the briefing was too limited for the interview partners, or if they did not trust the briefing physician enough, third-party opinions from other physicians and medical staff, or information from the internet were obtained.

Only one interview partner used the internet to gain information, which according to the literature, can be attributed to the age of our interview partners. In research regarding older people and the internet, it was shown that while many older people are familiar with the advantages of the internet, it is rarely used by people older than 50 [25]. The interview partners in the present investigation cannot be counted among the “off-liners” though, because half of them explicitly mentioned using the internet, particularly for e-mail.

Overall, women who did not participate in the medicinal study had increased feelings of emotional stress, increased fear of side effects and increased skepticism towards taking medication. Participating women argued with personal benefit, long-term protection and altruism. Furthermore the results of the interviews confirm literature emphasizing that the quality of communication between physician and patient is an important influential factor for decision-making. The interview partners indicated the quality of the briefing as essential reason for participation/non-participation. This applies to both the manner in which the conversation is led as well as the time spent having the conversation [9], [11], [26], [27].

In order to guarantee a decision-making process that is adequate for patients, existing standards for briefings have to be adhered to. Only in this manner can trust in the attending physicians be strengthened. Among others, the checklist by Brown et al. 2011, which in an adapted form could be used as a base for such a conversation, should be mentioned as suitable for this purpose [28].

In conclusion, it has to be noted that, through the present qualitative data collection, primarily reaction typologies of those interviewed were established. These reaction typologies can, among other things, be summarized in the following hypotheses (which are to be verified by quantitative studies):

- Mistrust towards physicians, studies and medication still exists.
- To be able to arrive at the “right” decision is, to an extremely high degree, dependent upon the communication and empathy abilities of the attending physicians.
- The recommended guidelines with regards to physician/patient communication, in practice, are not applied to their full extent.

## Supporting Information

Text S1 **Leading Interview Questions.**
(DOCX)
