# Supplementary material for: Insecurities of Women Regarding Breast Cancer Research: A Qualitative Study
Source: PLoS One. 2013 Dec 2;8(12):e81770. doi: 10.1371/journal.pone.0081770 (PMC3847121; doi:10.1371/journal.pone.0081770)
Supplement: Text S1 — Leading Interview Questions. (DOCX) [file pone.0081770.s001.docx]

# **Text S1: Leading Interview Questions**

- Why did you decide to participate/not participate in the study? What personal reasons do you have? *(Opening question).*
- Were you afraid of possible side effects with the study medication? What do you think of it, especially with regards to your willingness to participate?
- What do you think of the course of the study and the duration of the study? Do these items perhaps also have an influence on your decision to have participated/not participated in the study?
- Have you heard of the principle of randomization or random assignment in the context of the study? What do you think of it? Are you familiar with the term placebo? How do you feel about the use of a placebo in the study? (Explanation: placebo = substance without effect).
- Can you still remember your briefing regarding the study? How was it back then when the study was explained to you? Did you lack any information at that time that perhaps only occurred to you afterwards?
- In what personal health conditions were you at the time when you found out about the study? How were you doing?
- At the time of being invited to participate, what could potentially have prevented you from participating / what could possibly have encouraged you to participate?
- Is there perhaps anything else with regards to the study in question that I have not asked you and which you would like to tell me about? *(Final question)*
